# Cross-Sectional Analysis of Data from the U.S. Clinical Trials Database Reveals Poor Translational Clinical Trial Effort for Traumatic Brain Injury, Compared with Stroke

**DOI:** 10.1371/journal.pone.0084336

**Published:** 2014-01-08

**Authors:** Lucia M. Li, David K. Menon, Tobias Janowitz

**Affiliations:** 1 Division of Medicine, Imperial College London, London, United Kingdom; 2 Division of Anaesthesia, Cambridge University Hospitals NHS Foundation Trust, Cambridge, United Kingdom; 3 Department of Oncology, Cambridge University Hospitals NHS Foundation Trust, Cambridge, United Kingdom; Weill Cornell Medical College, United States of America

## Abstract

Traumatic brain injury (TBI) is an important public health problem, comparable to stroke in incidence and prevalence. Few interventions have proven efficacy in TBI, and clinical trials are, therefore, necessary to advance management in TBI. We describe the current clinical trial landscape in traumatic brain injury and compare it with the trial efforts for stroke. For this, we analysed all stroke and TBI studies registered on the US Clinical Trials (www.clinicaltrials.gov) database over a 10-year period (01/01/2000 to 01/31/2013). This methodology has been previously used to analyse clinical trial efforts in other specialties. We describe the research profile in each area: total number of studies, total number of participants and change in number of research studies over time. We also analysed key study characteristics, such as enrolment number and scope of recruitment. We found a mismatch between relative public health burden and relative research effort in each disease. Despite TBI having comparable prevalence and higher incidence than stroke, it has around one fifth of the number of clinical trials and participant recruitment. Both stroke and TBI have experienced an increase in the number of studies over the examined time period, but the rate of growth for TBI is one third that for stroke. Small-scale (<1000 participants per trial) and single centre studies form the majority of clinical trials in both stroke and TBI, with TBI having significantly fewer studies with international recruitment. We discuss the consequences of these findings and how the situation might be improved. A sustained research effort, entailing increased international collaboration and rethinking the methodology of running clinical trials, is required in order to improve outcomes after traumatic brain injury.

## Introduction

Traumatic Brain Injury (TBI) and stroke both have major public health impacts. They are common causes of brain injury, which present acutely and result in long-term personal and social consequences. Both change lives in an instant. Together, they account for 51% of mortality from central nervous system disease [1], [2]. The US prevalence for stroke and TBI are 6.8 million and 5.3 million, and the annual incidence are 800 000 and 1.6 million for stroke and TBI respectively [3]–[6]. Both conditions, but TBI in particular, are associated with a wide range of physical, cognitive and psychological deficits, which persist for years after injury [7]–[13]. Importantly, the age of onset in TBI and stroke is different. Half of all TBI events occur in people of working age (20–65 years old), which results in a longer burden of disability [3]. The cognitive and psychological deficits are also particularly detrimental in this age group because of the associated negative impact on return to previous educational or employment status [11]–[17]. Additionally, behavioural and psychiatric sequelae after TBI can have major and long-lasting disruptive effects on family relationships, with family members reporting high levels of psychological distress and cessation of work to provide care [18]–[21]. TBI consequently has a significant financial public health burden. Health economic estimates suggest that TBI accounts for over $70 billion (range $48–72 billion) in direct (e.g. long-term burden of medical care) and indirect (e.g. loss of productivity in a working population) costs in the USA annually [4], [22], [23], which is comparable to the estimated annual costs of stroke (range $38–74 billion) [24], [25].

These similarities between stroke and TBI in public health relevance are not reflected in evidence based treatment options or clinical trial efforts. Stroke translational research, with evidence from large-scale clinical trials, has resulted in changes in practice and improvements in outcome. The recent ‘FAST’ public health campaign in the UK has used billboard posters and TV adverts to promote rapid recognition of stroke by family members [26], thus enabling patients to reach hospital more quickly. The importance of timing in stroke treatment is driven by the tight timeframe required for thrombolysis, which has proven to be one of the most effective acute stroke treatment in recent years [6], [27]–[32].Thus, clinical research influences clinical practice, patient outcomes and government policy. On the other hand, the management of TBI remains based on mostly Level 2 and 3 evidence [33], with many putative neuroprotective strategies failing to realise their potential when translated from animal into human studies [34]. For instance, while therapeutic hypothermia showed promise in animal models, early human studies did not find conclusive evidence of benefit in TBI [35]–[37]. However, this intervention is currently the subject of a large multi-centre RCT, which has integrated experience of previous trials into protocol development [36]. The experience in stroke demonstrates what can be achieved when clinical interest is backed by an international research infrastructure. In order to achieve a high quality evidence base for clinical treatment in TBI that is comparable to stroke, substantial, high quality, relevant clinical trials are required.

This analysis examines the clinical research effort in TBI over the last decade, and compares it with stroke. The analysis focuses on key desirable study characteristics, and explores what lessons the neurotrauma clinical research community could learn from other clinical areas and new research paradigms.

## Materials and Methods

This is an observational, cross-sectional study of all clinical studies in TBI and stroke which were registered on the Clinical Trials Database (www.clinicaltrails.gov) between January 2000 and January 2013, inclusive. This analysis was conducted and reported according to the internationally agreed STrengthening the Reporting of OBservational studies in Epidemiology (STROBE) criteria [38]. The ClinicalTrials.gov database is the largest study database, and was chosen because of its size, ease of searching and capability to download data in a readily analysable format. Previous studies have used this database successfully to analyse study characteristics [39]–[42] and to compare study characteristics in different diseases.

The ‘Advanced Search’ function was used with the following terms “Stroke” and “Traumatic Brain Injury” with results being time-limited to 01/01/2000 to 01/31/2013. All studies listed under “Stroke” and “Cerebrovascular Disease” were also downloaded to ensure all potentially relevant stroke studies were obtained. In order to account for the very wide range of classifications for TBI, all studies listed under the following Topic categories were also downloaded: “Craniocerebral Injury”, “Chronic Brain Injury”, “Brain Hemorrhage, traumatic”, “Cerebral haemorrhage, traumatic”, “Brain concussion”, “Post-traumatic epilepsy” and “Brain Injuries”. Study details were downloaded as datasets and analysed in Microsoft Excel. All studies were manually checked for duplicates and suitability for inclusion. Studies were excluded if recruitment occurred outside of the specified analysis period, if the pathology investigated was not stroke or TBI (e.g. infantile ischaemia encephalopathy), or if the study population included non-stroke and non-TBI neurological disease (e.g. studies investigating intracranial hypertension as a result of any acute brain injury).

The registered enrolment number was manually checked for all studies whose registered status was ‘Completed’. The study title was used as a search string in both PubMed and Google Scholar and any publications found were used to verify actual enrolment numbers. Approximately one-third of studies could be verified in this way.

Studies classified as ‘Interventional’ (ClinicalTrials.gov Category: Study Types) were analysed further. This classification includes all studies where an intervention of any type (including drugs, procedures, rehabilitation strategies etc.) was investigated and distinguishes these studies from purely observational studies. Table 1 summarises how the studies were subsequently sub-classified. The full database and online registration entry was manually checked for any study which had missing information on the downloaded summary.

**Table 1:** Categorisation of study characteristics.

Characteristic	Registered label	Categories for analysis
Study Designs	Randomised, Non-Randomised	Randomised, Non-Randomised
Age Groups	Child	Paediatric
	Adult, Adult|Senior	Adult
	Child|Adult, Child|Adult|Senior	Both
Interventions	Categories were: ‘Drug’, ‘Device’, ‘Behaviour’, ‘Procedure’, ‘Other’ followed by a freetext description	Drug (includes blood products, intravenous fluid preparations)
		Device (includes non-invasive brain stimulation)
		Cognitive/Behaviour therapies
		Non-cognitive/behavioural therapies e.g. physiotherapy, treadmill training
		Procedure (includes surgical procedures)
		Protocol (includes educational measures)
Severity (TBI studies only)	n/a	Manual checking in inclusion criteria
Phases (only analysed for drug studies)	0, 1, 2, 3, 4, more than one phase noted	0, 1, 2, 3, 4, mixed phase
Location (manual checking of each study webpage)	n/a	Single Centre
		Multi-centre (same country)
		International

## Results

**Figure 1:**
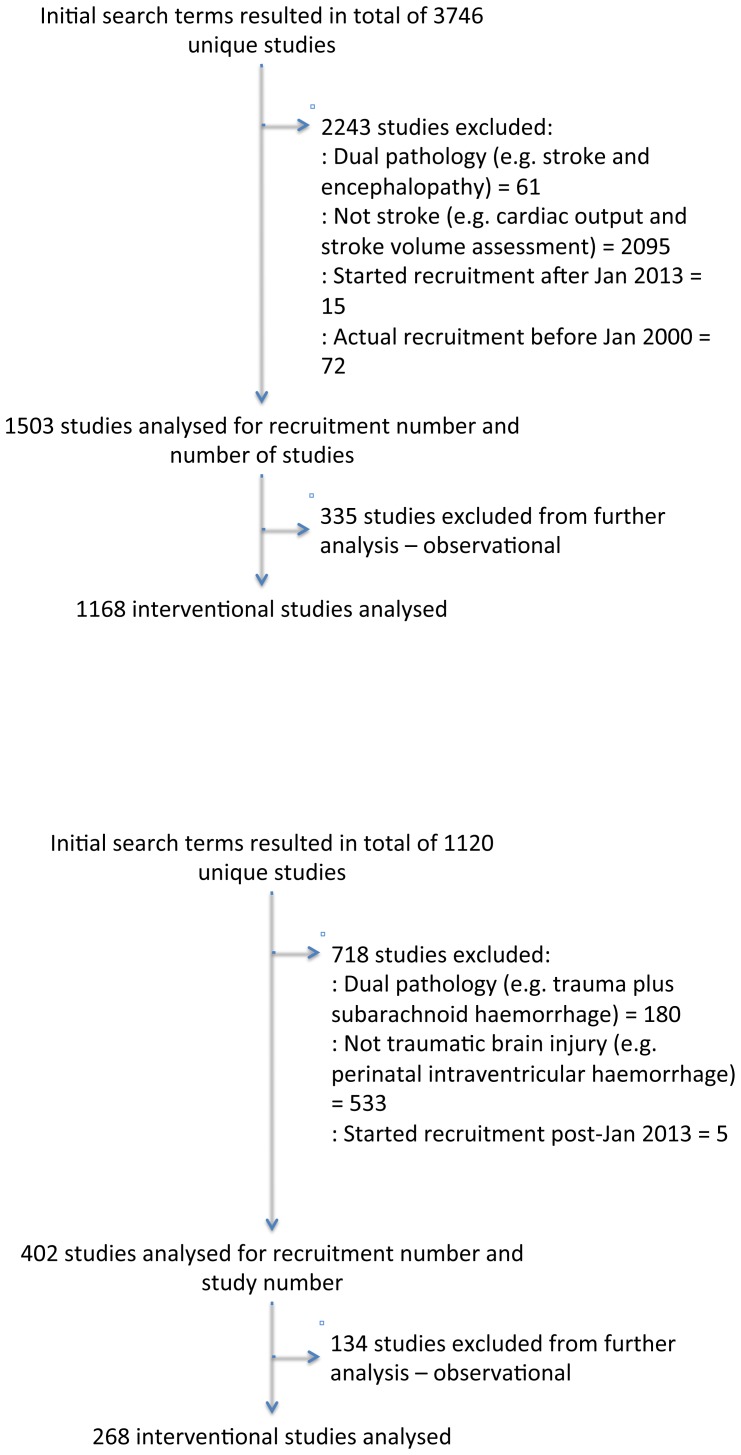
Flowchart of study inclusion for and exclusion from analysis for a) stroke studies and b) TBI studies.

The initial search criteria revealed 3716 unique studies in stroke and 1125 unique studies in TBI. We excluded 2156 stroke studies and 722 TBI studies from further analysis. Descriptive data of the enrolment for these studies are presented below and study characteristics are presented for the 1168 stroke and 268 TBI interventional studies found within the completed search (Figure 1).

### The relative number of clinical studies in TBI does not reflect its relative public health impact

**Figure 2:**
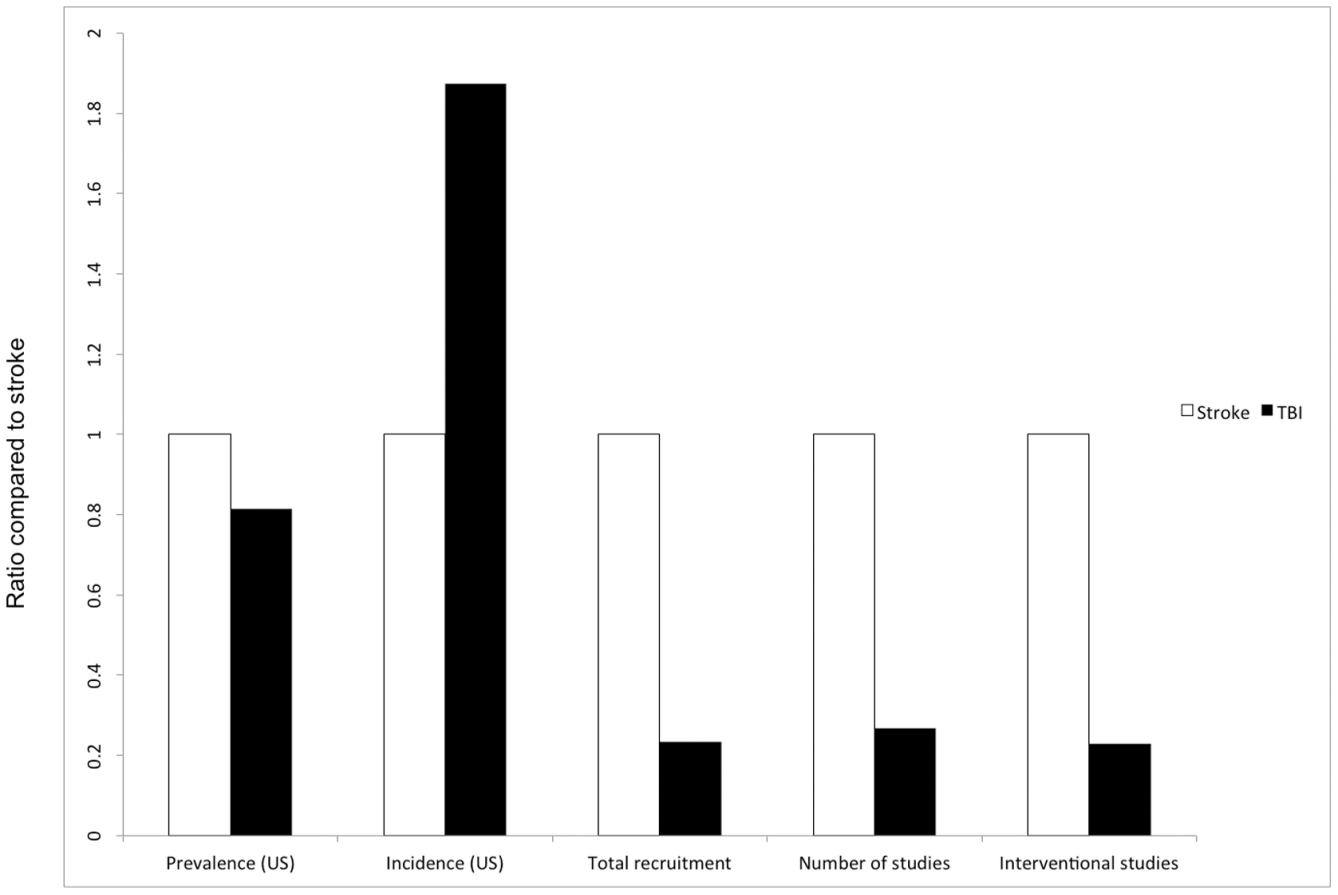
Key characteristics of TBI epidemiology and research as compared to stroke. The data for TBI are presented as a ratio of the stroke data. TBI has a similar prevalence and higher incidence when compared to stroke, but total recruitment numbers and number of studies are significantly fewer than in stroke.

The research effort in stroke is substantially greater than TBI, as measured by the total participant enrolment number (stroke n = 1 953 349 vs TBI n = 456 517), the total number of studies (stroke n = 1503 vs TBI n = 402) and the number of interventional studies (stroke n = 1168 vs TBI n = 268) (Fig. 2).

### The growth of TBI research lags behind the growth in stroke research

**Figure 3:**
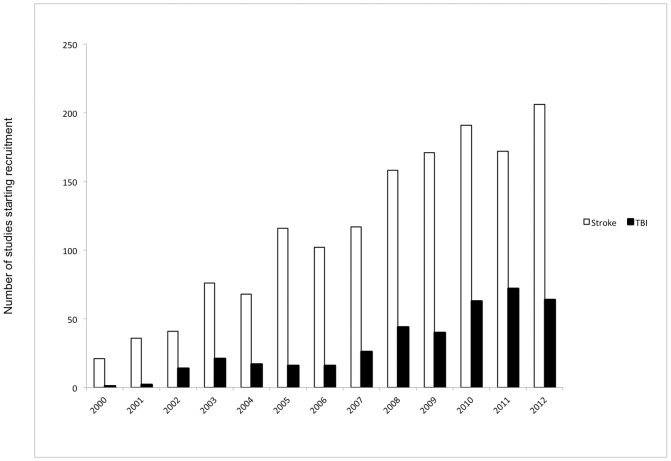
Number of studies starting recruitment in each year for stroke and TBI. Stroke has a greater increase in the number of studies starting recruitment annually as compared to TBI.

The number of studies starting recruitment per year has increased over the last 12 years for both stroke and TBI. However, there has been a much slower growth rate for TBI studies, with an average of 14 new studies per year in stroke, compared with only 5 new studies per year for TBI (Fig. 3).

### Studies with small recruitment numbers form the majority of both stroke and TBI studies

**Figure 4:**
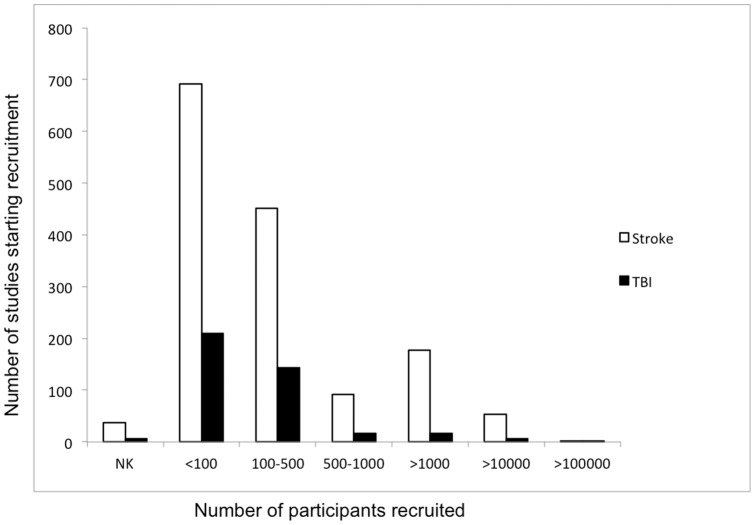
Number of studies per enrolment interval. The vast majority of both stroke (84%) and TBI (94%) studies recruit fewer than 1000 participants. NK = not known.

The majority of studies in both stroke and TBI have small recruitment numbers. Overall, 84% of stroke studies and 94% of TBI studies have a projected or actual recruitment of fewer than 1000 participants, with a median (interquartile range) or subject numbers of 100 (36–328) and 90 (34–180) respectively. Forty-seven percent of stroke studies and 53% of TBI studies recruit fewer than 100 participants (Fig. 4). These findings are consistent with those previously reported for studies within this database [39].

### The majority of stroke and TBI studies are interventional studies

Interventional studies make up 77% (n = 1168) and 66% (n = 268) of total stroke and TBI studies registered on this database respectively. Sixty percent (n = 1 168 311) [95% CI: 62–58%] of participants in stroke studies and 79% (n = 359 482) [95% CI: 75–82%] in TBI studies are enrolled in interventional studies. Randomised control trials make up the vast majority of both interventional TBI (81% n = 958) and stroke (82% n = 217) studies.

The largest group of interventional studies are drug studies for both stroke (44%) and TBI (57%) (Table 2). TBI has a much higher proportion of cognitive/psychological therapies (stroke 2.3% vs TBI 19.5%).

**Table 2:** Number of interventional studies investigating each type of intervention.

Intervention	Stroke	TBI
Drug	512	151
Device	273	20
Cognitive/Behavioural	27	52
Non cognitive/behavioural therapies	230	12
Procedure	58	16
Protocol	51	13
Mixed	17	4

For drug trials, Trial Phase information was only available in 69% (n = 801) of stroke studies and in 65% (n = 185) of TBI studies. Of those studies in which Trial Phase information was available, stroke research had 13 Phase 0, 99 Phase I, 215 Phase II, 215 Phase III, 143 Phase IV and 115 mixed phase trials whereas TBI research had 2 Phase 0, 24 Phase I, 50 Phase II, 41 Phase III, 42 Phase IV and 26 mixed phase trials.

### Single-centre studies dominate in both TBI and stroke research

**Figure 5:**
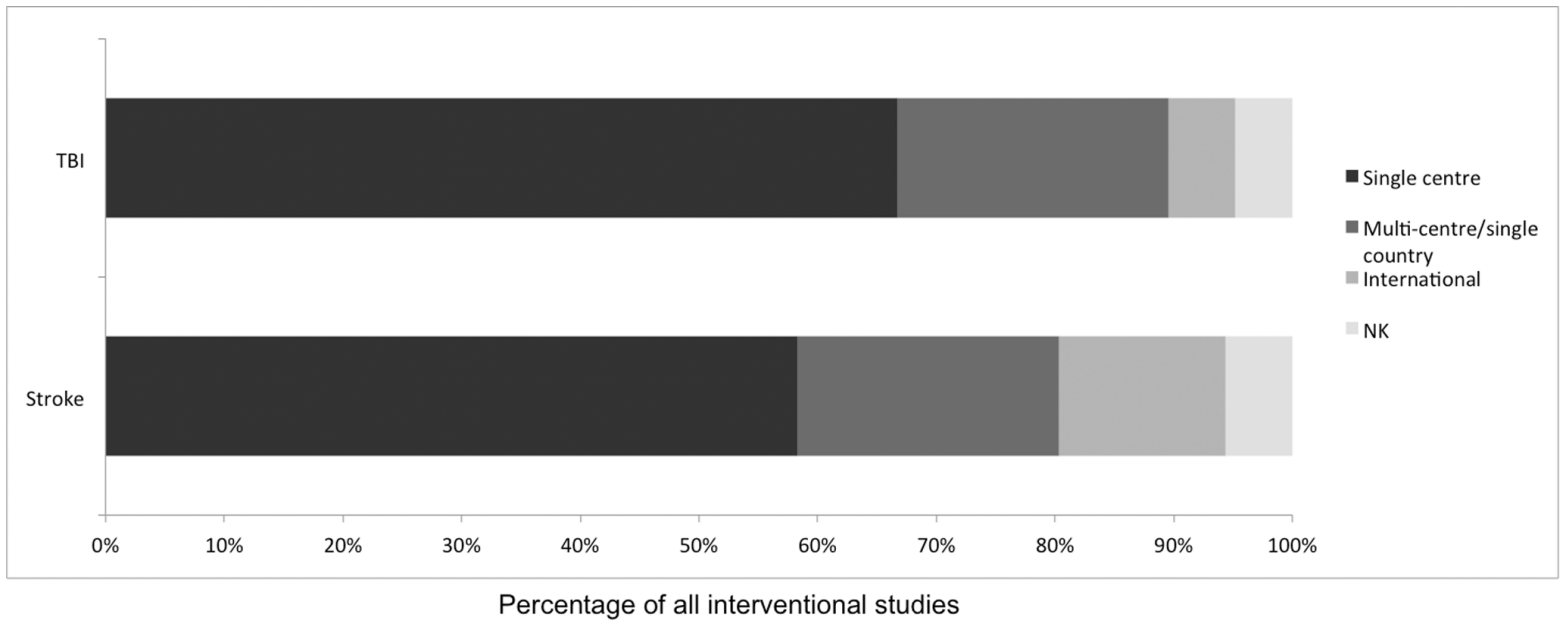
Figure depicts location of study recruitment. The majority of both stroke (58%) and TBI (67%) studies are single centre. NK = not known.

The majority of interventional studies for both stroke and TBI recruit from single centres with international studies representing both a minority of studies (stroke n = 164 vs TBI n = 15) and participants (stroke n = 565 320 [48%, 95% CI: 45–51%] vs TBI = 5105 [1.4%, 95% CI: 0–3%]). However, stroke has a much higher percentage of international studies (stroke 15% [95% CI: 13%–17% vs TBI 6% [95% CI: 3%–9%]) (Figure 5).

### Interventional studies in TBI recruit across all age groups and severity types

TBI studies have a higher proportion which investigate children (4% [n = 12] exclusively recruiting children and 23% [n = 62] recruiting both children and adults) as compared with stroke studies (1% [11] exclusively recruiting children and 8% recruiting all ages [82]).

The majority of interventional TBI studies recruit moderate-severe TBI patients (87.3%, n = 234). Forty-one percent (n = 111) of interventional TBI studies recruit only moderate/severe TBI patients. Thirty-seven percent (n = 100) also recruited mild TBI patients but all recruited patients had evidence of deficit, for example, impaired performance in cognitive assessments. A fifth of studies (20.5%, n = 55) recruited mild TBI patients but did not specify whether a specific deficit needed to be present.

## Discussion

This study investigated quantitatively the clinical studies registered on the ClinicalTrials.gov database in the fields of stroke and TBI with regard to study characteristics.

We compared in particular the following characteristics between TBI and stroke clinical research: recruitment number, study count, and trends in annual study registration. These parameters greatly influence the likelihood of practice changing results from clinical trial efforts.

Overall, the research effort in stroke, as measured by number of studies and patient recruitment, is significantly greater that in TBI. This discrepancy is in contrast to the comparative public health impact of these two diseases as measured by incidence, prevalence, and cost [3], [4]. This mismatch is particularly stark when compared to the research profile within other specialties. For example, a recent review of oncological clinical trials registered in ClinicalTrials.gov demonstrated a positive correlation between number of trials conducted into each cancer type and its associated public health impact (incidence and mortality) [42].

The majority of clinical trials in both stroke and TBI were drug trials. Although surgical interventions have been trialled and are now commonly used in ischaemic stroke [43], such interventions are much more commonly used, and should hence be trialled, in TBI. Given the absence of a clear commercial motivation, trials of surgical intervention are mostly funded by public grants. In most cases, surgical therapies will evaluate the relative merit of one existing intervention over another in an attempt to provide an evidence base for current surgical practice. An additional difference for trials of surgical intervention is that they are not driven by the stringent and restrictive regulatory framework which exists for trials of new medicinal products [44]. Development of a new drug, on the other hand, requires substantial financial investment prior to phase III trials. Given the long list of negative clinical trials of presumed neuroprotective agents, there is now considerable reluctance amongst companies to proceed along this path. As a result, most TBI drug trials currently investigate new licensing indications for already available drugs and do so with public funding.

Level 1 evidence can only be obtained from sufficiently powered randomised controlled trials (RCTs). Multi-centre and multi-ethnicity recruitment also results in better generalizability of results. An encouraging finding is that the majority of clinical trials in both stroke and TBI are RCTs. However, only 6% of trials in TBI recruit internationally compared to 15% in stroke. In addition, while the last decade has seen a rise in clinical trial activity for both conditions, the rate of increase is higher for stroke and the absolute difference in trial activity between stroke and TBI is consequently larger each year.

For both conditions, stroke and TBI, the majority of studies are small (around half recruit fewer than 100 patients) and the overwhelming majority of both stroke and TBI studies recruit fewer than 1000 patients. The small recruitment numbers may partly be due to the desire to recruit as homogenous a study population as possible. However, it is likely that many of the smaller studies will be under-powered. The implications of this are both clinical (since the results will not be informative for clinical practice) and scientific (since under-powered studies overestimate effect size, which will negatively influence the planning of further studies) [45]. Additionally, a large number of small trials will reduce the number of patients that consent to large, multi-centre trials and this will further hinder the development of successful interventions.

Recruitment is a particular difficulty for TBI studies, and this is likely to be an important reason for the abundance of small-scale studies. Some of this is attributable to the ethics of recruiting TBI patients in the acute period where they are unable to consent. Research without consent is not a readily accepted idea [46] but TBI clinical trials have protocols to enable recruitment, for example, consent from family members with renewal of consent from patients if they later regain capacity. However, by far the biggest reason for recruitment difficulties is due to the pathological and clinical heterogeneity of the TBI population. For example, the DECRA trial screened 3478 severe TBI patients but excluded only 21 for consent issues [47], [48]. The large majority were excluded because the TBI did not have the characteristics needed for the trial. On the other hand, a recent drug trial of minor stroke/transient ischaemic attacks excluded patients primarily for protocol reasons, for example, delayed presentation [48]. The heterogeneity of TBI disadvantages TBI research in the traditional clinical trial format,

Therefore, the research effort into TBI not only needs to increase in quantity, but also needs to alter its approach. Adaptations to the traditional RCT design, in particular Comparative Effectivness Research (CER) and Multi-Arm Multi-Stage (MAMS) studies, may be particularly useful in TBI [49], [50].

A key concept of CER is that of Pragmatic trials, where the effectiveness of an intervention is evaluated for real-world patient populations and healthcare conditions [49]. This would enable broader applicability of results. Another aspect of CER is the combination of many data sources, observational as well as interventional studies; this could enable the results of smaller-scale studies to be combined in meta-analyses that are more likely to yield practice-changing conclusions. One other important concept of CER is that of sub-population analysis; this would increase recruitment as the inclusion criteria could be broader and, if appropriately powered, heterogeneities in the trial population are turned into an advantage.

A MAMS trial uses a single control group with several parallel intervention arms (the ‘multiple arms’). This saves on the number of patients required since only one control group is required. Interim analyses are performed to identify interventions which are likely to be ineffective; these are terminated early and, where possible, the participants in these arms crossover into the ongoing intervention arms (the ‘multiple stages’). This increases the recruitment number in each of the final interventions which increases the trial's power [50]. MAMS design has been successfully adopted in cancer trials [50]–[52]. However, both MAMS and CER designs would help to maximise the efficiency of recruitment as each enrolled participant has a higher chance of receiving an effective intervention and, as a consequence, the resulting studies have greater power. Encouraging collaboration through international research networks along with consensus statements among clinicians on efforts in TBI trials are crucial factors for enabling CER and MAMS trials. Strengthening such networks will additionally improve the capacity for performing large-scale, multi-centre trials.

As well as trial design, there are other relevant issues when considering the future of TBI research. TBI is traditionally classified according to severity. Severe TBI, though representing the minority (<10%) of total TBI [53], has the highest associated relative mortality and morbidity, so it is unsurprising that the majority of TBI studies recruit severe TBI. However, we included all TBI studies in our comparison because there is a growing realisation that ‘mild’ TBI, even those not initially requiring medical attention, can result in long-term neuropsychological consequences. The American Academy of Neurology has recently published a consensus statement on managing concussion at the sidelines of contact sport games, with the view that these impacts can result in long-term damage [54]. Veterans of the Iraq/Afghan wars sustaining blast injuries, which had been classified as ‘mild’ TBIs, can have long-term cognitive deficits [55]. People in professions exposing them to mild head injuries also appear to sustain cognitive consequences along with biological evidence of neuronal damage [56]. Therefore, given that even a mild ‘TBI’ can lead to cognitive consequences, and that cognitive consequences are a major influence on outcome [57], the study of mild TBI is an important future area of research.

Attention to the finer details of methodology is also important to improve the quality of TBI trials. It is particularly crucial to use appropriate outcome measures when designing trials which will impact clinical practice. The outcomes recorded in trials must be important to patients e.g. return to work, level of independence, as well as healthcare providers e.g. mortality, use of healthcare resources. This is a particular challenge for TBI trials for several reasons. First, heterogeneity in pre-morbid characteristics and mechanism and extent of injury produces a vast number of different deficits. These range from physical signs e.g. limb weakness to physical symptoms e.g. headaches and dizziness, from cognitive e.g. concentration and attentional difficulties to neuropsychiatric e.g. irritability. Second, ‘secondary deficits’ can also occur and have a significant impact on recovery e.g. seizures can result in reduced independence through loss of the ability to drive. Third, the common methods of synthesising the impact of these many heterogenous deficits have problems of their own. Outcome ‘batteries’ and combined scoring systems often have no particular logic reported for the relative weighting of each component. Generic quality of life scores e.g. GOS/GOS-E, SF-36, QUOLIBRI-TBI all have their own biases. Fourth, many of the outcome measures important to patients (e.g. return to work/education), whilst pragmatic, are very insensitive due to their myriad components. For example, an intervention to improve concentration may be very effective but the patient is still unable to return to their previous work as a bus driver because they have seizures. Thus, within the confines of available outcomes measures, appropriate selection and powering is crucial. Fifth, patients' self-evaluation of their recovery may not truly reflect their recovery e.g. due to loss of insight, due to depression [58]. In these situations, carer evaluation may be helpful [59].

The success of clinical trials in TBI is, of course, dependent on the validity of the underlying hypothesis for the intervention. New trials, therefore, must be based on pre-clinically tested and verified interventions. Assumptions, particularly from pre-clinical drug studies on animal models, should be critically evaluated for their applicability to ‘real life’ TBI before they are allowed to influence study design [34].

A final point to consider is that of funding. Stroke and TBI both result in significant burdens to the individual and society. To put this into context, cancer, of any type, has an annual incidence of approximately 320 000 in the UK, as compared with 150 000 for stroke and 135 000 for TBI requiring hospital admission [60]–[63]. In 2012, the main charities for specialty research funding in the UK spent £332 million on cancer research, compared with £4 million for stroke and less than £100 000 for TBI research [63]–[65] (charity annual accounts). Whilst charitable funding for TBI research also comes from other sources in the UK, it is unlikely to amount to more than £10million per annum. This means that TBI receives disproportionately little funding. This will impact on the type of trials which can be performed since high-quality, multi-centre, large-scale trials require substantial funding.

### Limitations

There are two main limitations of our study.

First, we used a single database. Not all clinical trials may be registered on ClinicalTrials.gov [66], and the widening of this analysis to other databases, for example the World Health Organisation portal, would provide more security that the analysis was complete. This may be a particular issue for small scale observational studies, since there are no regulatory or publication requirements to register these. Thus, the number of observational studies found on the ClnicalTrials.gov database may well be an underestimate. However, the second part of our analysis relies on interventional studies, where the registration bias may be less relevant. Analysis of the chosen database has been employed by previous studies and there is consensus that the ClinicalTrials.gov database includes a representative majority of clinical trials [39]–[42].

Second, entry of information into this database is by investigators of sponsors from the trial team and our analyses inevitably depends on the quality of data recorded. Although there are automated and manual procedures in place to improve the accuracy and relevance of the information available (e.g. reminders to the researchers), there is no mandated requirement to update entries [67]. We attempted to minimise some of these issues by manually checking some fields e.g. enrolment numbers.

Third, a direct comparison between TBI and stroke clinical trial research is limited by the significant variability within TBI severuty, which does not characterise stroke to the same extent. Many cases of ‘mild’ TBI, unless they occur within a specified setting, such as contact sports, do not receive specialist medical attention. On the other hand, most ‘minor’ stroke/transient ischaemic attack patients will receive prompt specialist medical attention. Thus, there is likely to be a selection bias against ‘mild’ TBI research which limits the usefulness of a direct TBI and stroke research database comparison. This bias can be expected to improve with growing clinical recognition of ‘mild’ TBI and with new study designs which can adequately analyse the entire spectrum of TBI disease.

## Conclusions

Analysis of the trials registered on the ClinicalTrials.gov database demonstrates substantial differences between the research effort into stroke and TBI. This is even more concerning when considering the gap in the context of other disease burdens. This gap is in sharp contrast to the equivalent public health importance of the two diseases. Furthermore, trial registration statistics of recent years indicate that this imbalance is likely to worsen further in favour of stroke in the years to come. However, it is not simply enough to increase the number of studies, if their design does not allow them to actually answer important questions. Instead, TBI translational research must learn from other diseases and specialties, such as stroke, cardiovascular disease, and oncology, where major clinical advances have been made through large-scale, multi-centre trials studying interventions based robust preclinical data and hypotheses. Collaborative efforts within the research community, improved funding structures and thinking beyond the traditional clinical trial format will improve both the quantity and quality of clinical trial research into TBI.
